# Using sniffer dogs to enhance bedbug detection and environmental management in a university hospital

**DOI:** 10.1016/j.infpip.2026.100562

**Published:** 2026-06-19

**Authors:** H. Watanabe, T. Kobayashi, A. Okugawa, H. Fujita, Y. Watanabe, I. Nakamura

**Affiliations:** Department of Infection Control and Prevention and Department of Infectious Diseases, Tokyo Medical University Hospital, Tokyo, Japan

**Keywords:** Bedbugs, Sniffer dogs, Hospital environmental management, Environmental monitoring, Infection control

## Abstract

**Background:**

Bedbug incidents in healthcare facilities are uncommon, and standardized management protocols are generally lacking.

**Local problem:**

In 2018, bedbugs were unintentionally introduced into our university hospital through an admitted patient’s personal belongings, and 10 bedbugs were identified on the bed, wall, and floor.

**Methods (Intervention):**

Following the Ministry of Health, Labour and Welfare’s ‘Guidelines for Hygiene Management in the Hotel Industry’, we implemented carpet replacement and disinfestation by a specialized company. To ensure complete eradication, we additionally used trained sniffer dogs for odour-based detection as a complementary intervention.

**Results:**

Sniffer dog detection successfully identified no remaining bedbugs after conventional disinfestation and proved useful as an adjunctive method for confirming eradication. The intervention required modest additional costs related to staffing and environmental decontamination.

**Conclusions:**

The use of sniffer dogs may serve as a practical and cost-conscious tool for early identification and confirmation of bedbug eradication in healthcare settings, underscoring the importance of preventive environmental design and early detection strategies.

## Introduction

Carpeted flooring was commonly used in hospitals during the 1980s, when comfort and aesthetic considerations were often prioritized over infection control principles [1]. This trend was also observed in Japan. When our university hospital was built in 1985, infection control measures were not emphasized as strongly as they are today, and carpeted flooring was installed to create a more luxurious atmosphere.

Bedbugs prefer narrow, dark crevices such as gaps between walls or the underside of carpets, although they are vulnerable to light and heat. Their larvae measure approximately 1.5 mm, whereas adult bedbugs are 5–7 mm in size and visible to the naked eye [2,3]. A female adult lays five to six eggs per day, producing a total of 200–500 eggs over its lifetime, and the eggs hatch in about two weeks, making infestations difficult to control [2,4].

In Japan, bedbug infestations had increasingly been reported in the hotel industry, prompting the Ministry of Health, Labour and Welfare to issue recommendations such as the ‘Essential Hygiene Practices for the Hotel Business’ [5] and the guidance ‘On the Implementation of Pest Control Measures in Hotels’ [6]. Given these characteristics, the carpeted flooring in our hospital provided a favourable environment for bedbugs. Over time, this became a persistent issue that interfered with medical services.

The incident described in this report occurred in 2018, during the final years before our hospital relocated to a new facility in 2019, where the flooring was replaced with easy-to-clean materials. Thus, the bedbug problem emerged in the context of an ageing building with carpeted flooring and limited environmental control measures.

To address this local problem, we sought to determine whether bedbugs persisted in patient-related areas even after conventional disinfection and cleaning. We implemented sniffer dog–based detection as a complementary method to enhance the reliability of bedbug eradication.

## Methods

### Intervention: sniffer dog–assisted detection

Trained sniffer dogs, typically beagles, were deployed in pairs for each inspection. While one dog conducted the search, the other remained calm in a cage on standby. Each dog wore a protective cover to prevent contamination of the hospital environment. At each inspection site, the active dog systematically examined the area and detected the odour associated with bedbugs or their eggs. The survey was conducted more than two weeks after environmental cleaning and carpet replacement, considering the approximate hatching period of bedbug eggs.

### Study of the intervention

When the active sniffer dog indicated a positive reaction at a site, it was removed from the area, and the second dog was brought in to confirm the finding. If the second dog demonstrated the same reaction at the same location, the presence of bedbugs or their eggs was considered likely. Inspection sites were selected based on patient movement, environmental characteristics, and the potential for bedbug harbouring within the affected areas.

### Measures

The primary outcome was the presence or absence of sniffer dog alerts at each inspection site. Alerts were categorized into three predefined levels:

• **Strongly suggestive:** both dogs alerted at the same location
• **Weakly suspicious:** only one dog alerted
• **Negative:** neither dog alerted

### Analysis

Findings were summarized descriptively according to alert category and location. No quantitative statistical analysis was performed as the purpose of the survey was to confirm eradication rather than to compare outcomes across groups.

## Results

After environmental cleaning and carpet replacement were completed, a sniffer dog survey was conducted more than two weeks after the incident, considering the approximate hatching period of bedbug eggs. Five locations were inspected:

1. a special independent air-conditioned room on the first floor
2. the visitors’ shower room in the first-floor emergency department
3. the inpatient room on the 10th ward floor
4. the general waiting area on the second floor
5. a consultation room in the outpatient area on the second floor

No reactions were observed in locations 1, 2, 3, or 4.

Weakly suspicious reactions were detected in three areas:

• the patient long bench in the pre-consultation waiting space (dog A)
• the patient chair in the consultation room (dog B)
• the bedside wall sheet in the consultation room (dog B)

**Figure 1:**
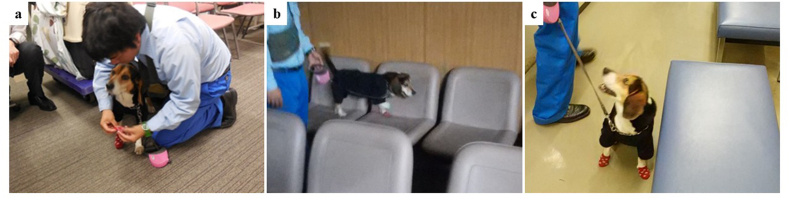
Sniffer dog preparation and environmental exploration. a. Preparation of the sniffer dog, including the application of protective foot covers. b. Exploration of the general waiting area, including the sofa. c. Reaction to the patient long chair in the preconsultation waiting space.

No site showed a positive reaction by both sniffer dogs; therefore, no location was classified as strongly suggestive. The procedures for sniffer dog preparation and environmental exploration are illustrated in Figure 1. A summary of the sniffer dog reactions at each site is presented in Table I.

**Table I:** Sniffer dog reactions at each survey location and corresponding classification

Survey location	Dog A	Dog B	Classification
Special independent air-conditioned room	(−)	(−)	Negative
Visitors’ shower room	(−)	(−)	Negative
Inpatient room	(−)	(−)	Negative
General waiting area – sofa	(−)	(−)	Negative
Pre-consultation waiting space – patient long chair	(+)	(−)	Weakly suspicious
Consultation room – patient bed	(−)	(+)	Weakly suspicious
Consultation room – bedside wall sheet	(−)	(+)	Weakly suspicious

(−), no reaction; (+), reaction.

## Discussion

### Summary

Bedbug incidents can occur not only in the hotel industry but also within healthcare facilities, where they represent an environmental sanitation issue rather than an infectious disease concern [7]. Because hospitals generally lack an established management framework for dealing with bedbug infestations, our institution also had no prior experience with such an event. The Department of Infection Control and Prevention, together with the hospital management division, therefore assumed responsibility for addressing the situation.

### Interpretation

Several contextual factors may have contributed to the incident. The architectural design of our hospital included carpeted flooring, which provided a favourable environment for bedbugs. In addition, the hospital was located near a downtown area of Tokyo with numerous low-budget lodging facilities and a homeless shelter – settings known to be at high risk for bedbug infestation. A survey in southern France reported infestations in 13% of shelter rooms [8], and practical reports highlight that shared sleeping areas, high turnover, and limited sanitation resources facilitate persistent transmission [9]. Similar sanitation issues, including bedbugs, have also been documented in shelters in the USA [10]. These surrounding conditions may have increased the likelihood of bedbug introduction into the hospital.

Because no hospital-specific guidelines exist, our management approach was adapted from the Ministry of Health, Labour and Welfare’s recommendations for the hotel industry [5,6]. A critical aspect of management was the need to account for the approximately two-week hatching period of bedbug eggs, which required ongoing monitoring even after cleaning and disinfection.

Odour-based detection is an important component of continuous monitoring because bedbugs and their eggs emit characteristic odours. Sniffer dogs have an extensive track record in odour detection tasks, including forensic and drug-related investigations, and have recently been applied in the medical field to detect infectious diseases [11,12] and certain cancers [13] through disease-specific volatile organic compounds. In this case, we employed sniffer dogs that had received specialized training to detect odours associated with bedbugs and their eggs.

### Limitations

This approach has several limitations. Because sniffer dogs are living animals, their performance may be influenced by factors such as physical condition and environmental factors during the search. To minimize this limitation, searches were conducted using a pair of dogs, allowing one dog to confirm the findings of the other. In addition, the total direct cost of the response – including carpet replacement, disposal of beds and related items, room disinfection, disposal and cleaning of outpatient furniture where the sniffer dogs reacted, and the sniffer dog service fees – was approximately 1.3 million Japanese yen. One inpatient bed had to be closed for two weeks, resulting in an additional opportunity cost not included in the direct expenditure. Therefore, the actual financial impact was likely higher than the calculated amount.

### Ethical considerations

In addition to these methodological limitations, we also addressed ethical and operational considerations related to the use of sniffer dogs. The dogs deployed in this project were trained working dogs provided by a professional pest-control company that routinely conducts bedbug detection in hotels and other facilities. Their work–rest cycles, health monitoring, and behavioral management were overseen entirely by professional handlers in accordance with the company's established welfare protocols, consistent with recommendations in the medical detection dog literature [14].

To minimize physical and psychological stress, the dogs worked in short intervals with appropriate rest periods, and two dogs operated as a pair, alternating their detection tasks to avoid excessive workload. After each session, handlers performed routine checks to confirm the dogs’ physical condition and readiness for further activity.

During hospital deployment, the dogs were transported on a large cart while remaining inside their dedicated transport containers, which reduced vibration and environmental stimuli and helped maintain a low-stress environment. The dogs were always accompanied by handlers, did not have direct contact with patients, and moved only through predetermined routes that avoided major human traffic. Importantly, detection activities in outpatient areas were conducted exclusively on hospital holidays, when no patients were present, ensuring both safety and minimal disruption. When the dogs were taken out of their transport containers for active detection work, they wore protective suits and protective foot covers to ensure both animal safety and hospital hygiene.

### Implications for practice

None of the sites showed reactions from both sniffer dogs. For the three locations where only one dog reacted, additional cleaning and replacement of surface materials were performed. Our hospital has since relocated to a new facility with hard flooring, which is easier to clean and less conducive to bedbug infestation. Although hard flooring is generally preferable for bedbug management, certain infestations or residual odours may still be difficult to identify through visual inspection alone. In this context, sniffer dogs remain a valuable complementary tool for detecting signs that are not readily apparent.

In conclusion, We reported our experience using sniffer dogs as part of the countermeasures against bedbugs in a hospital setting. For hospital flooring, materials that are easy to clean – rather than carpet – are desirable from both infection control and pest management perspectives. Bedbug incidents can occur even in healthcare facilities, and odour-based detection using trained sniffer dogs represents a valuable tool for early identification and management. The financial burden associated with bedbug control can be substantial, underscoring the importance of preventive environmental design and early detection strategies. Our experience suggests that sniffer dogs can serve as a practical complementary method for detecting signs that are not readily identifiable through visual inspection alone, supporting their potential role in hospital environmental management.

## CRediT authorship contribution statement

**H. Watanabe:** Writing – review & editing, Writing – original draft, Visualization, Validation, Supervision, Project administration, Methodology, Investigation, Conceptualization. **T. Kobayashi:** Writing – review & editing, Validation, Investigation. **A. Okugawa:** Writing – review & editing, Visualization, Validation, Investigation. **H. Fujita:** Writing – review & editing, Validation, Investigation. **Y. Watanabe:** Writing – review & editing, Validation, Investigation. **I. Nakamura:** Writing – review & editing, Supervision, Methodology.

## Ethical approval

This report describes an operational bedbug inspection conducted as part of routine hospital management. No patient data were collected, and no human subjects were involved; therefore, ethical approval was not required.

## Declaration of generative AI and AI-assisted technologies in the writing process

During the preparation of this manuscript, the authors used Microsoft Copilot (Microsoft Corporation, Redmond, WA, USA) to support language refinement, organization of content, and improvement of clarity. After using this tool, the authors carefully reviewed, edited, and verified all content to ensure accuracy, comprehensiveness, and impartiality. The authors take full responsibility for the content of the published article.

## Funding sources

This research received no external funding.

## Conflict of interest statement

The authors declare no conflicts of interest.
